# Construction and validation of machine learning models for predicting distant metastases in newly diagnosed colorectal cancer patients: A large‐scale and real‐world cohort study

**DOI:** 10.1002/cam4.6971

**Published:** 2024-03-16

**Authors:** Ran Wei, Guanhua Yu, Xishan Wang, Zheng Jiang, Xu Guan

**Affiliations:** ^1^ Department of Colorectal Cancer Surgery, National Cancer Center/National Clinical Research Center for Cancer/Cancer Hospital Chinese Academy of Medical Sciences and Peking Union Medical College Beijing China; ^2^ Department of Gastrointestinal Surgery, The First Affiliated Hospital Sun Yat‐sen University Guangzhou Guangdong China

**Keywords:** artificial intelligence, colorectal cancer, distant metastases, machine learning, risk stratification

## Abstract

**Background:**

More accurate prediction of distant metastases (DM) in patients with colorectal cancer (CRC) would optimize individualized treatment and follow‐up strategies. Multiple prediction models based on machine learning have been developed to assess the likelihood of developing DM.

**Methods:**

Clinicopathological features of patients with CRC were obtained from the National Cancer Center (NCC, China) and the Surveillance, Epidemiology, and End Results (SEER) database. The algorithms used to create the prediction models included random forest (RF), logistic regression, extreme gradient boosting, deep neural networks, and the K‐Nearest Neighbor machine. The prediction models' performances were evaluated using receiver operating characteristic (ROC) curves.

**Results:**

In total, 200,958 patients, 3241 from NCC and 197,717 CRC from SEER were identified, of whom 21,736 (10.8%) developed DM. The machine‐learning‐based prediction models for DM were constructed with 12 features remaining after iterative filtering. The RF model performed the best, with areas under the ROC curve of 0.843, 0.793, and 0.806, respectively, on the training, test, and external validation sets. For the risk stratification analysis, the patients were separated into high‐, middle‐, and low‐risk groups according to their risk scores. Patients in the high‐risk group had the highest incidence of DM and the worst prognosis. Surgery, chemotherapy, and radiotherapy could significantly improve the prognosis of the high‐risk and middle‐risk groups, whereas the low‐risk group only benefited from surgery and chemotherapy.

**Conclusion:**

The RF‐based model accurately predicted the likelihood of DM and identified patients with CRC in the high‐risk group, providing guidance for personalized clinical decision‐making.

## 1 INTRODUCTION

Colorectal cancer (CRC) is the third most common malignant tumor worldwide, with the number of CRC patients expected to reach approximately 2.5 million by 2035.^1^, ^2^ Distant metastases (DM) are present in 20% of the patients newly diagnosed with CRC. Although great improvements have been made in the treatment of CRC, metastatic CRC remains a fatal disease that leads to half of all CRC‐related deaths.^3^ DM from CRC severely impairs survival outcomes and the average survival of patients with metastatic CRC is only 30 months.^4^, ^5^, ^6^ The most common site of CRC metastasis is the liver, followed by the lungs. Thus, chest and abdominal computed tomography (CT) is recommended for detecting hepatic and pulmonary metastases. Metastases of CRC to the bones and brain are relatively rare. The incidence of bone metastases in CRC patients varies from 6.0% to 10.4%.^7^, ^8^ However, the prognosis for bone metastases is poor. The 5‐year survival rate of patients with bone metastases is <5%, and the median survival of these patients is <7 months.^9^, ^10^ Owing to the low incidence and asymptomatic nature of bone metastases, bone imaging is often ignored in clinical practice. Until patients with CRC present with symptoms of metastasis‐induced bone destruction, such as skeletal‐related events, diagnostic imaging tests are suggested for bone metastasis localization. Thus, CRC patients with bone metastases may miss optimal therapeutic opportunities.^11^ Similar to bone metastases, brain metastases are rare events that typically occur later in the course of CRC. The incidence of brain metastases from CRC varies from 0.6% to 3.2%.^12^, ^13^, ^14^, ^15^ Despite their low incidence, brain metastases from CRC progress aggressively and have a poor prognosis; the median survival after the diagnosis of brain metastases is <8 months.^16^, ^17^ Presently, the diagnosis of brain metastases mainly depends on neurological symptoms, and routine neurological imaging is not recommended for patients newly diagnosed with CRC.^18^ Given the poor prognosis for DM in patients with CRC, a more precise model for predicting DM is needed.

As a vital aspect of artificial intelligence, machine learning has performed well in diagnosing and predicting multiple diseases and has exhibited higher accuracy than conventional methods in clinical settings.^19^, ^20^ With the rapid development of algorithms, machine learning has been used to predict the likelihood of DM in patients diagnosed with solid cancers by analyzing high volumes of complex heterogeneous clinical data.^21^, ^22^, ^23^ However, no machine‐learning‐based model has been developed to predict the possibility of DM in patients newly diagnosed with CRC. Therefore, this study was designed to establish novel machine learning‐based models to predict the risk of CRC metastases to the liver, lung, bone, and brain using clinicopathological data from the National Cancer Center (NCC, Beijing, China) and the Surveillance, Epidemiology, and End Results (SEER) database. This could help clinicians promptly detect DM and select appropriate treatment strategies to improve prognosis. Furthermore, risk stratification based on this risk prediction model was created to classify patients newly diagnosed with CRC into different groups according to the risk of DM, for predicting the prognosis and treatment response of patients with metastatic CRC and help clinicians select the optimal treatment.

## 2 MATERIALS AND METHODS

### 2.1 Data source and study population

This retrospective cohort study collated the clinicopathological data from the period January 1, 2010 to December 31, 2018 of CRC patients from the SEER database. These anonymized data were categorized into a training set and a test set. The external validation set from the period January 1, 2010 and December 31, 2018 was obtained from the Electronic Medical Record System of the National Cancer Center/National Clinical Research Center for Cancer/Cancer Hospital of the Chinese Academy of Medical Sciences, and the Peking Union Medical College (Beijing, China). The ethics committee of the Cancer Hospital, Chinese Academy of Medical Sciences, approved this study (NCC2019S‐060). Written informed consent for retrospectively analyzing the anonymized data was not required.

### 2.2 Main outcomes and selected clinicopathological features

**FIGURE 1:**
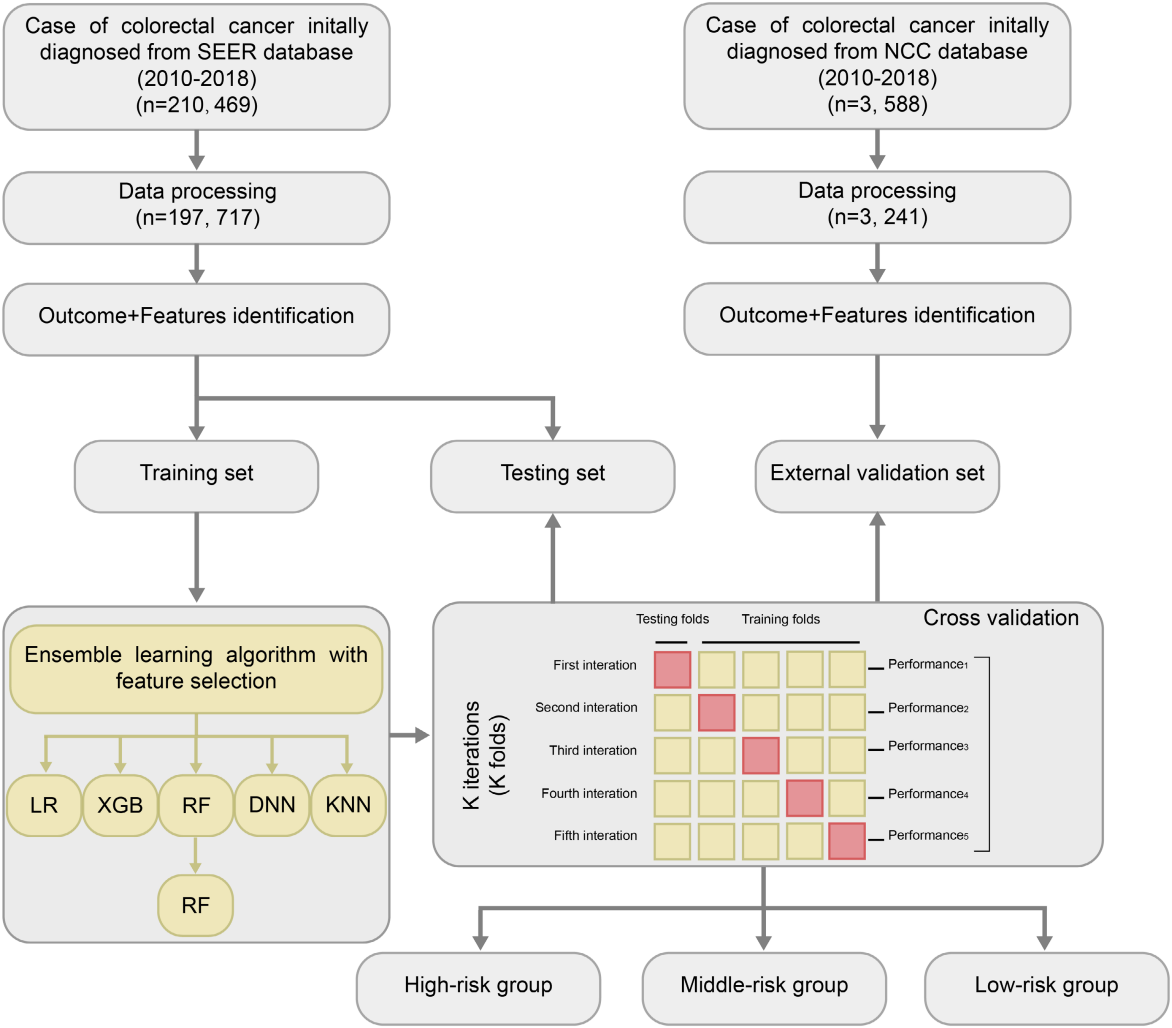
Flow diagram of the study population. In this study, a total of 197,717 CRC patients from SEER database were included, which were divided into the independent training and independent test sets in a ratio of 8:2, and 3241 patients from NCC were included in external validation set. The predictive models and risk stratification were established to help provide reliable individual information for CRC treatment recommendations. CRC, colorectal cancer; DNN, deep neural network; KNN, K‐Nearest Neighbor; LR, logistic regression; NCC, National Cancer Center; RF, random forest; SEER, Surveillance, Epidemiology, and End Results; XGboost, extreme gradient boosting.

This cohort study included patients diagnosed with primary CRC. The clinical outcome was the presence of DM due to CRC, including CRC metastases to the liver, lungs, bones, and brain. The diagnosis of cancer was based on topographic or histological classification, following the International Classification of Diseases for Oncology‐3 (ICD‐O‐3)/World Health Organization 2008 guidelines. The American Joint Committee on Cancer (AJCC) 6th edition and SEER combined stage (2016+) TNM staging were used in our study. The exclusion criteria were as follows: (1) unknown metastatic status at initial diagnosis, (2) unknown pathohistological diagnosis, (3) age < 20 years, (4) diagnosis of benign or borderline tumors, and (5) lack of complete data on treatment and clinicopathological characteristics. Demographic and tumor characteristics were collected, and this process is shown in Figure 1.

### 2.3 Feature engineering and data transformation

To create prediction models, feature engineering approaches were used to process the easily accessible clinicopathological data from the SEER database and the Electronic Medical Record System of the NCC. We used cross‐validation (CV) and recursive feature elimination iteratively to filter variables using a random forest (RF) classifier to increase the accessibility of the prediction models. Cross‐validation was used for internal validation as a reliable method to monitor the development of machine learning and enhance the performance of the models. The variables were evaluated based on their relative significance in the receiver operating characteristics (ROC) of the prediction models.

### 2.4 Development of risk models and risk stratification

CRC patients drawn from the SEER database were randomly separated into training and test sets in an 8:2 ratio, and those from the NCC populated the external validation set. The risk models for predicting the likelihood of developing DM were built into the training set and were subsequently assessed and verified in the test and external validation sets. The RF, logistic regression (LR), extreme gradient boosting (XGboost), deep neural network (DNN), and K‐Nearest Neighbor (KNN) algorithms were developed by performing a 10‐fold CV in the training set. Variables that were strongly associated with the probability of DM were identified using univariate and multivariate logistic regression analyses. The mutual relationships between the variables incorporated in this study were analyzed using correction analysis. These prediction models were built and validated using the “caret” and “gbm” packages.

Our preliminary findings indicated that the performances of these various machine learning methods for DM prediction were essentially similar. However, the DeLong test showed that on the training, test, and external validation sets RF exhibited a tendency toward improved availability. Further to quantify the risk of DM developing in patients with CRC, we formulated a risk score for each patient using the RF model and based thereon ranked the patients from high to low. The risk scores were calculated with RF algorithm based on the age at CRC diagnosis, race, gender, year at diagnosis, tumor histology, tumor location and size, T‐stage, N‐stage, tumor grade, harvested lymph nodes, and primary tumor, and every patient had a private risk score. A higher risk score means potentially higher risk for distant metastases and vice versa. According to risk scores of each CRC patient, which were ranked from high to low, these CRC patients were separated into three risk groups, with about 65,906 (33.3%) patients in each group. Those with the highest risk scores were assigned to the high‐risk group, whereas those with the lowest risk scores were assigned to the low‐risk group.

### 2.5 Statistical analysis

The Mann–Whitney U test was used to compare differences across continuous variables, whereas the chi‐squared test was used for categorical variables. In the survival analysis, the Kaplan–Meier method and log‐rank test were used to determine the prognostic differences between different risk groups. The CRC patients, whether undergoing therapy or not (including surgery, chemotherapy, and radiotherapy), were divided into 1:1 groups using Propensity Score Matching (PSM). To evaluate the performance of the different models, sensitivity, specificity, Gini coefficient, the area under the ROC curve (AUC), and 95% confidence intervals (CIs) were generated based on the number of correctly recognized true‐positive instances and the number of incorrectly categorized false‐positive instances. All analyses were performed using version 3.6.1 of R.

## 3 RESULTS

### 3.1 Clinicopathological characteristics

In total, data from 197,717 CRC patients from the SEER database and 3241 patients from the NCC database were used in this study (Figure 1). From the SEER database, 20,043 (11.3%) patients developed DM of the CRC, including the bone, brain, liver, and lungs. In total, 88,181 (45.0%) CRC patients >70 years old, 95,286 (48.2%) patients suffered from right colon cancer, 53,055 (26.8%) patients suffered from left colon cancer, 49,403 (25.0%) patients suffered from rectum cancer. One hundred thirty nine of 825 (70.7%) patients with CRC were at the T3/T4 stage and 85,142 (43.1%) were at the N1/N2 stage (Table S1). Additionally, those from the SEER database were randomly separated into a training set (*n* = 158,174) and a test set (*n* = 39,543) in an 8:2 ratio (Figure 1). Table 1 summarizes the clinicopathological characteristics of the patients enrolled in this study.

**TABLE 1:** Demographic and tumor characteristics of patients with colorectal cancer in SEER and NCC databases.

Characteristic	Training set from SEER			Test set from SEER			External validation set from NCC		
	Nonmetastasis (*n* = 142,047)	Metastasis (*n* = 16,127)	*p* value	Non‐Metastasis (*n* = 35,627)	Metastasis (*n* = 3916)	*p* value	Nonmetastasis (*n* = 1548)	Metastasis (*n* = 1693)	*p* value
Age at CRC diagnosis, No. (%), years			<0.001			<0.001			<0.001
20–49	14,818 (10.4)	2556 (15.8)		3787 (10.6)	612 (15.6)		476 (30.8)	369 (21.8)	
50–69	62,209 (43.8)	8044 (49.9)		15,541 (43.6)	1969 (50.3)		839 (54.2)	1109 (65.5)	
≥ 70	65,020 (45.8)	5527 (34.3)		16,299 (45.8)	1335 (34.1)		233 (15.0)	215 (12.7)	
Gender, No. (%)			<0.001			<0.001			<0.001
Female	69,248 (48.8)	7024 (43.6)		17,427 (48.9)	1784 (45.6)		737 (47.6)	638 (37.7)	
Male	72,799 (51.2)	9103 (56.4)		18,200 (51.1)	2132 (54.4)		811 (52.4)	1055 (62.3)	
Year of CRC diagnosis, no. (%)			<0.001			<0.001			0.003
2010–2013	70,373 (49.5)	8373 (51.9)		17,721 (49.7)	2073 (52.9)		682 (44.1)	658 (38.9)	
2014–2018	71,674 (50.5)	7754 (48.1)		17,906 (50.3)	1843 (47.1)		866 (55.9)	1035 (61.1)	
Race, *n* (%)			<0.001			<0.001			1.000
White	113,015 (79.6)	12,232 (75.8)		28,384 (79.7)	3001 (76.6)		0 (0.0)	0 (0.0)	
Black	15,922 (11.2)	2390 (14.8)		3882 (10.9)	590 (15.1)		0 (0.0)	0 (0.0)	
Other	13,110 (9.2)	1505 (9.4)		3361 (9.4)	325 (8.3)		1548 (100.0)	1693 (100.0)	
Grade, no. (%)			<0.001			<0.001			<0.001
Grade 1/2	116,022 (81.7)	11,707 (72.6)		29,150 (81.8)	2835 (72.4)		934 (60.3)	1205 (71.2)	
Grade 3/4	26,025 (18.3)	4420 (27.4)		6477 (18.2)	1081 (27.6)		614 (39.7)	488 (28.8)	
Primary tumor site, no. (%)			<0.001			<0.001			<0.001
Right colon	69,134 (48.7)	7086 (43.9)		17,363 (48.7)	1703 (43.5)		446 (28.8)	391 (23.1)	
Left colon	37,444 (26.3)	4956 (30.7)		9386 (26.4)	1269 (32.4)		348 (22.5)	107 (6.3)	
Rectum	35,469 (25.0)	4085 (25.4)		8878 (24.9)	944 (24.1)		754 (48.7)	1195 (70.6)	
T stage, no. (%)			<0.001			<0.001			<0.001
T1/T2	44,516 (31.3)	1664 (10.3)		11,308 (31.7)	404 (10.3)		679 (43.9)	95 (5.6)	
T3/T4	97,531 (68.7)	14,463 (89.7)		24,319 (68.3)	3512 (89.7)		869 (56.1)	1598 (94.4)	
N stage, no. (%)			<0.001			<0.001			<0.001
N0	86,437 (60.9)	3569 (22.1)		21,732 (61.0)	837 (21.4)		1371 (88.6)	317 (18.7)	
N1/N2	55,610 (39.1)	12,558 (77.9)		13,895 (39.0)	3079 (78.6)		177 (11.4)	1376 (81.3)	
Tumor histology, no. (%)			<0.001			<0.001			<0.001
Adenocarcinomas	97,817 (68.9)	12,896 (80.0)		24,602 (69.1)	3126 (79.8)		1312 (84.8)	1604 (94.7)	
Other	44,230 (31.1)	3231 (20.0)		11,025 (30.9)	790 (20.2)		236 (15.2)	89 (5.3)	
Tumor size, no. (%)			<0.001			<0.001			<0.001
(0–2)	25,544 (18.0)	794 (5.0)		6532 (18.3)	201 (5.2)		77 (4.9)	828 (48.9)	
(2–5)	70,804 (49.8)	7778 (48.2)		17,811 (50.0)	1901 (48.5)		843 (54.5)	631 (37.3)	
>5	45,699 (32.2)	7555 (46.8)		11,284 (31.7)	1814 (46.3)		628 (40.6)	234 (13.8)	
Number of nodes examined, no. (%)			<0.001			<0.001			<0.001
>12	112,586 (79.3)	11,236 (69.7)		28,161 (79.0)	2711 (69.2)		99 (6.4)	829 (48.9)	
<12	29,461 (20.7)	4891 (30.3)		7466 (21.0)	1205 (30.8)		1449 (93.6)	864 (51.1)	
Surgery, no. (%)			<0.001			<0.001			<0.001
No	4217 (3.0)	2391 (14.8)		1076 (3.0)	567 (14.5)		844 (54.5)	165 (9.8)	
Yes	137,830 (97.0)	13,736 (85.2)		34,551 (97.0)	3349 (85.5)		704 (45.5)	1528 (90.2)	
Chemotherapy, no. (%)			<0.001			<0.001			<0.001
No	90,192 (63.5)	4780 (29.6)		22,839 (64.1)	1184 (30.2)		731 (47.2)	17 (1.0)	
Yes	51,855 (36.5)	11,347 (70.4)		12,788 (35.9)	2732 (69.8)		817 (52.8)	1676 (99.0)	
Radiation, no. (%)			0.029			0.024			<0.001
No	123,607 (87.0)	14,132 (87.6)		31,070 (87.2)	3465 (88.5)		1342 (86.7)	72 (4.3)	
Yes	18,440 (13.0)	1995 (12.4)		4557 (12.8)	451 (11.5)		206 (13.3)	1621 (95.7)	
Primary tumor, no. (%)			<0.001			<0.001			1.000
Yes	110,866 (78.0)	13,474 (83.5)		27,713 (77.8)	3254 (83.1)		1548 (100.0)	1693 (100.0)	
No	31,181 (22.0)	2653 (16.5)		7914 (22.2)	662 (16.9)		0 (0.0)	0 (0.0)	

*Note*: *p* values were calculated using the chi‐squared test for categorical variables.

Abbreviations: CRC, colorectal cancer; NCC, National Cancer Center; SEER, Surveillance, Epidemiology, and End Results.

### 3.2 Variable feature importance of DM prediction

Linear correlation analyses were performed using univariate and multivariate logistic regression to identify the risk features of the DM prediction models (Table 2). Univariate and multivariate logistic regression analyses suggested the following as risk factors for predicting DM: age at CRC diagnosis, sex, race, primary tumor site, tumor histology, tumor size, T stage, N stage, harvested lymph nodes, surgery, chemotherapy, and radiotherapy. The T3/T4 stage (adjusted OR: 1.52; 95% CI: 1.48–1.55), N stage (adjusted OR: 3.46; 95% CI:3.32–3.61), tumor grade (adjusted OR: 1.17; 95% CI: 1.13–1.22), tumor size (adjusted OR: 2.46; 95% CI: 2.27–2.66), number of nodes examined (adjusted OR: 1.92; 95% CI: 1.84–2.00), and chemotherapy (adjusted OR: 1.97; 95% CI: 1.89–2.04) were correlated with a higher risk of developing DM. Additionally, the age at CRC diagnosis (adjusted OR: 0.75; 95% CI: 0.71–0.79), nonadenocarcinomas (adjusted OR: 0.70; 95% CI: 0.67–0.73), primary tumor (adjusted OR: 0.91; 95% CI: 0.87–0.95), and surgery (adjusted OR: 0.59; 95% CI: 0.58–0.61) were correlated with a decreased risk of DM in CRC patients.

**TABLE 2:** Univariable and multivariable logistic regression analyses for patients with metastatic CRC in SEER database.

Characteristic	Univariable logistic regression		Multivariable logistic regression	
	HR (95% CI)	*p* value	HR (95% CI)	*p* value
Age at CRC diagnosis				
20–49	Ref		Ref	
50–69	0.76 (0.72–0.79)	<0.001	0.92 (0.87–0.96)	<0.001
≥70	0.50 (0.47–0.52)	0.001	0.75 (0.71–0.79)	<0.001
Gender				
Female	Ref		Ref	
Male	0.21 (1.17–1.24)	<0.001	1.17 (1.13–1.21)	<0.001
Race, No. (%)				
White	Ref		Ref	
Black	1.39 (1.34–1.46)	<0.001	1.32 (1.26–1.38)	<0.001
Other	1.02 (0.97–1.08)	0.429	0.92 (0.87–0.97)	0.002
Grade, No. (%)				
Grade1/2	Ref		Ref	
Grade3/4	1.71 (1.66–1.77)	<0.001	1.17 (1.13–1.22)	<0.001
Primary tumor site				
Right Colon	Ref		Ref	
Left Colon	1.31 (1.26–1.35)	<0.001	0.90 (0.86–0.93)	<0.001
Rectum	1.11 (1.07–1.15)	<0.001	0.48 (0.45–0.50)	<0.001
T stage				
T1/T2	Ref		Ref	
T3/T4	2.16 (2.12–2.21)	<0.001	1.52 (1.48–1.55)	<0.001
N stage				
N0	Ref		Ref	
N1/N2	5.68 (5.49–5.89)	<0.001	3.46 (3.32–3.61)	<0.001
Tumor histology				
Adenocarcinomas	Ref		Ref	
Other	0.55 (0.53–0.57)	<0.001	0.70 (0.67–0.73)	<0.001
Tumor size				
(0–2)	Ref		Ref	
(2–5)	3.86 (3.59–4.14)	<0.001	1.87 (1.73–2.01)	<0.001
>5	5.87 (5.47–6.31)	<0.001	2.46 (2.27–2.66)	<0.001
Number of nodes examined				
>12	Ref		Ref	
<12	0.68 (1.62–1.73)	<0.001	1.92 (1.84–2.00)	<0.001
Primary tumor				
Yes	Ref		Ref	
No	0.70 (0.67–0.73)	<0.001	0.91 (0.87–0.95)	<0.001
Surgery				
No	Ref		Ref	
Yes	0.67 (0.66–0.68)	<0.001	0.59 (0.58–0.61)	<0.001
Chemotherapy				
No	Ref		Ref	
Yes	4.13 (4–4.27)	<0.001	1.97 (1.89–2.04)	<0.001

*Note*: Logistic regression analysis was used to calculate the hazard ratio (HR) and 95% confidence interval (CI) based on metastatic CRC. Covariables that were significant in univariable logistic regression analysis (*p* < 0.05) are included in the multivariable analysis.

Abbreviations: CI, confidence interval; CRC, colorectal cancer; HR, hazard ratio; SEER, Surveillance, Epidemiology, and End Results.

### 3.3 Model performance

To create reliable and accurate predictive models, recursive feature elimination and 10‐fold‐CV were employed to select features iteratively based on the implementation of the RF classifier in the training set. Furthermore, to predict the likelihood of developing DM in newly diagnosed CRC patients before receiving treatment, and to help physicians select the optimal treatment for these patients, the features associated with the treatment (including surgery and chemoradiotherapy) were excluded from the prediction models. Twelve other features (age at CRC diagnosis, race, sex, year at diagnosis, tumor histology, tumor location and size, T‐stage, N‐stage, tumor grade, harvested lymph nodes, and primary tumor) were evaluated during the development of the machine learning‐based models.

**FIGURE 2:**
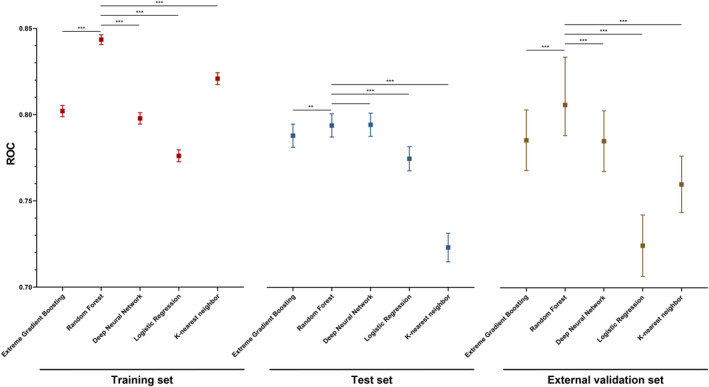
ROC values of postoperative complications prediction for the XGboost, RF, DNN, LR, and KNN in training set, test set, and external validation set. DeLong test; *p value <0.05, **p value <0.01, ***p value <0.001. DNN, deep neural network; KNN, K‐ Nearest Neighbor; LR, logistic regression; RF, random forest; ROC, receiver operating characteristic; XGboost, extreme gradient boosting.

**FIGURE 3:**
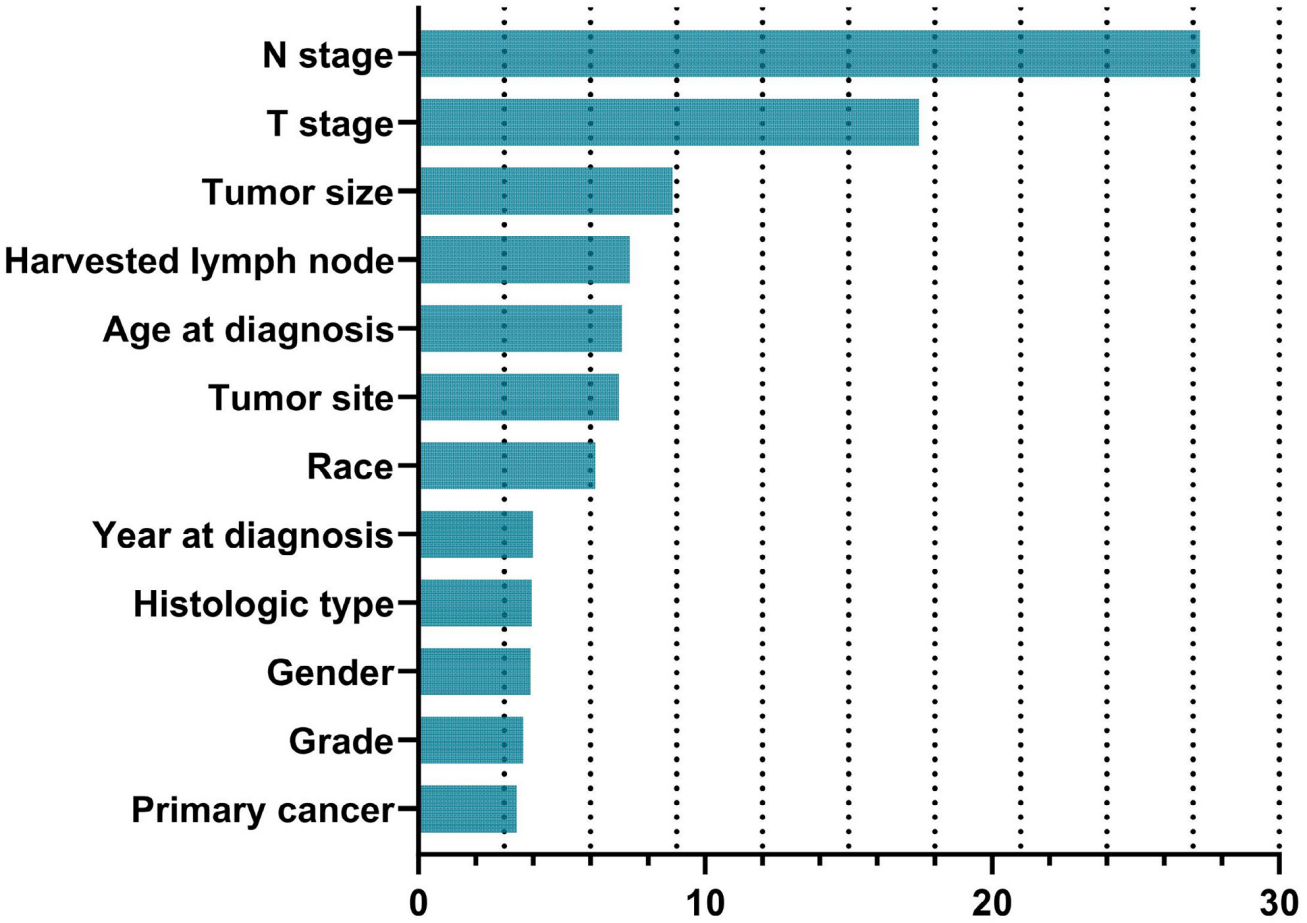
The feature importance for predicting postoperative complications of random forest.

To predict DM in CRC patients, five machine learning models based on the 12 aforementioned features were developed using the RF, LR, XGboost, DNN, and KNN algorithms. To evaluate the performance of the models, the AUC with *p* value and 95% confidence interval, specificity, sensitivity, and Gini score were calculated (Tables S2–S5). In comparison with the KNN (AUC = 0.820, 0.723, and 0.759), DNN (AUC = 0.776, 0.774, and 0.724), XGB (AUC = 0.802, 0.788, and 0.785), and LR (AUC = 0.797, 0.794, and 0.785) models, the findings revealed that the RF model exhibited highest accuracy on the training set (AUC = 0.843), test set (AUC = 0.793), and external validation set (AUC = 0.806). The DeLong test was performed to analyze the ROC of these risk models, which showed that the RF model substantially improved the ROC for predicting DM compared with the other risk models (Figure 2, Tables S3–S5). After analyzing the specificity, sensitivity, AUC, and Gini scores of all models, the RF model exhibited the best performance (Table S2). The sensitivities and specificities of the prediction models were identical. By comparing the gain values of the characteristic with the DM prediction, we assessed the significance of each (Tables S6–S10). Despite the different models exhibiting slight variances in the importance of features the findings of the overall results showed that in all models, the N stage and T stage were the most important risk factors. In the RF model, N stage, T stage, tumor size, and harvested lymph nodes were the most significant risk factors for predicting the likelihood of developing DM (Figure 3).

### 3.4 Risk stratification for patients

**FIGURE 4:**
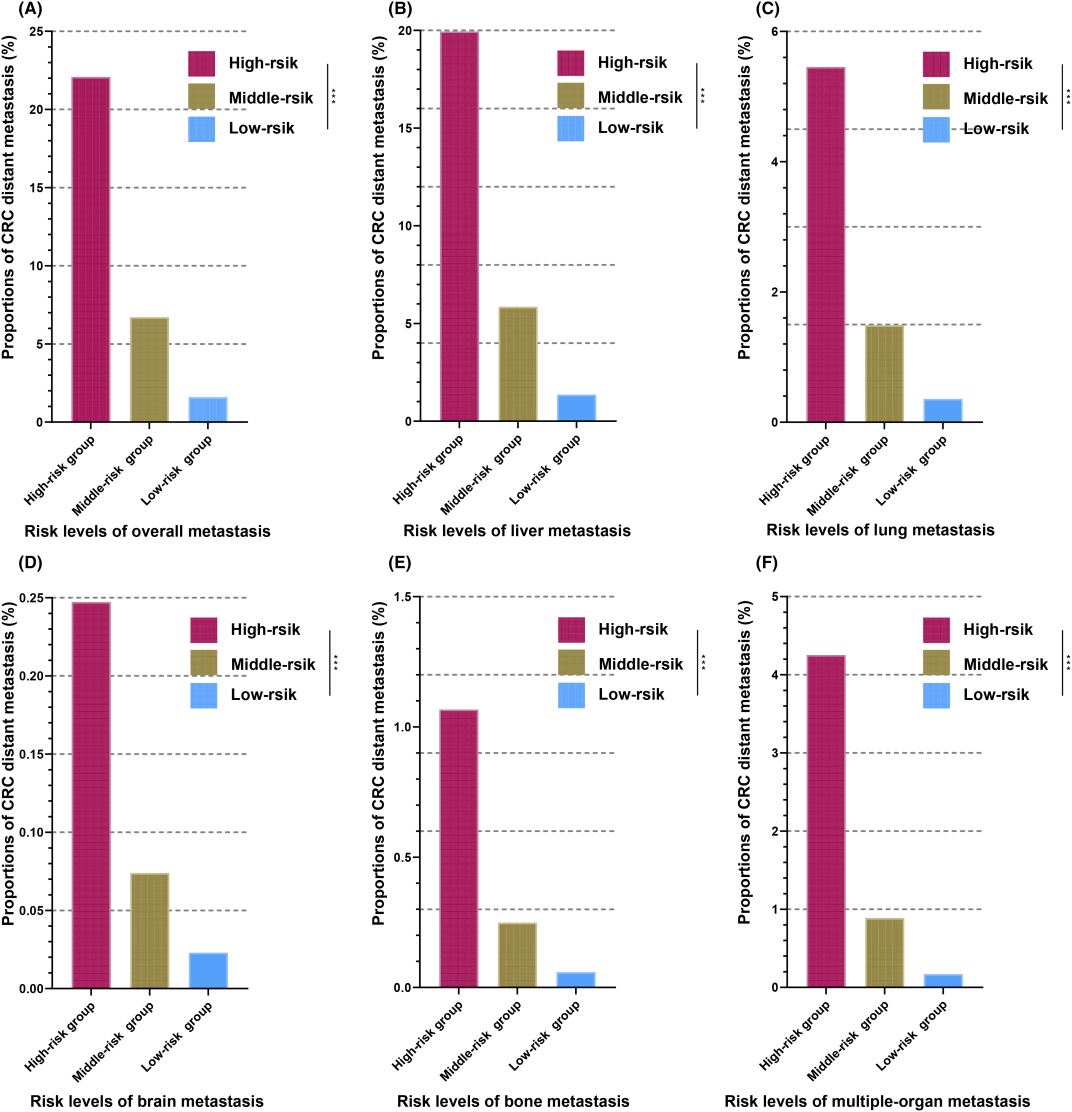
Risk levels for predicting distant metastasis of CRC using RF. The risk scores of developing overall distant (A), liver (B), lung (C), brain (D), bone (E), and multiple‐organ (F) metastasis based on RF. Sorted by risk scores form high to low, patients with CRC were divided into three risk groups of the same number: high‐risk, middle‐risk, and low‐risk groups. The distant metastasis rates were significantly higher in the high‐risk group than in other groups. (***p value < 0.001). CRC, colorectal cancer; RF, random forest.

**FIGURE 5:**
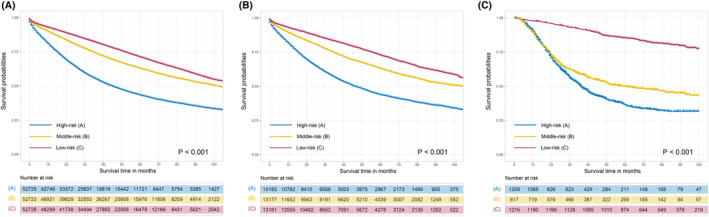
The survival comparison among high‐risk, middle‐risk, and low‐risk groups. The overall survival were significantly worse in high‐risk group than in middle‐risk group and low‐risk group in training set (A), test set (B), and external validation set (C).

We used an RF classifier to calculate the risk score for each CRC patient to predict their likelihood of developing DM. According to the risk scores of each CRC patient, which ranged from high to low, these CRC patients fell into three risk groups, with approximately 65,906 (33.3%) in each group (Figure 4A–F, Table S11). Those with the highest risk scores were assigned to the high‐risk group, whereas those with the lowest risk scores were assigned to the low‐risk group. The remaining patients were assigned to the middle‐risk group. In the high‐risk group, 14,553 (22.1%) patients developed overall metastases, 4426 (6.7%) patients in the middle‐risk group developed overall metastases, and 1064 (1.6%) patients in the low‐risk group developed overall metastases. The risk classification was also appropriate for the proportion of patients with single CRC metastases to the liver, lung, brain, and bone, suggesting that patients in the high‐risk group had the highest rates of DM and patients in the low‐risk group had the lowest DM risk. Regarding patients who developed multiorgan metastases, the high‐risk group had proportionally more than the other groups. Furthermore, we compared the 5‐year overall survival (OS) rates among the three groups (Figure 5A–C). The survival analysis demonstrated distinct differences in the survival probabilities among them. The CRC patients with the highest risk scores had the lowest OS rate, while patients in the low‐risk group had the highest OS rate.

### 3.5 Treatment benefits for three risk groups

**FIGURE 6:**
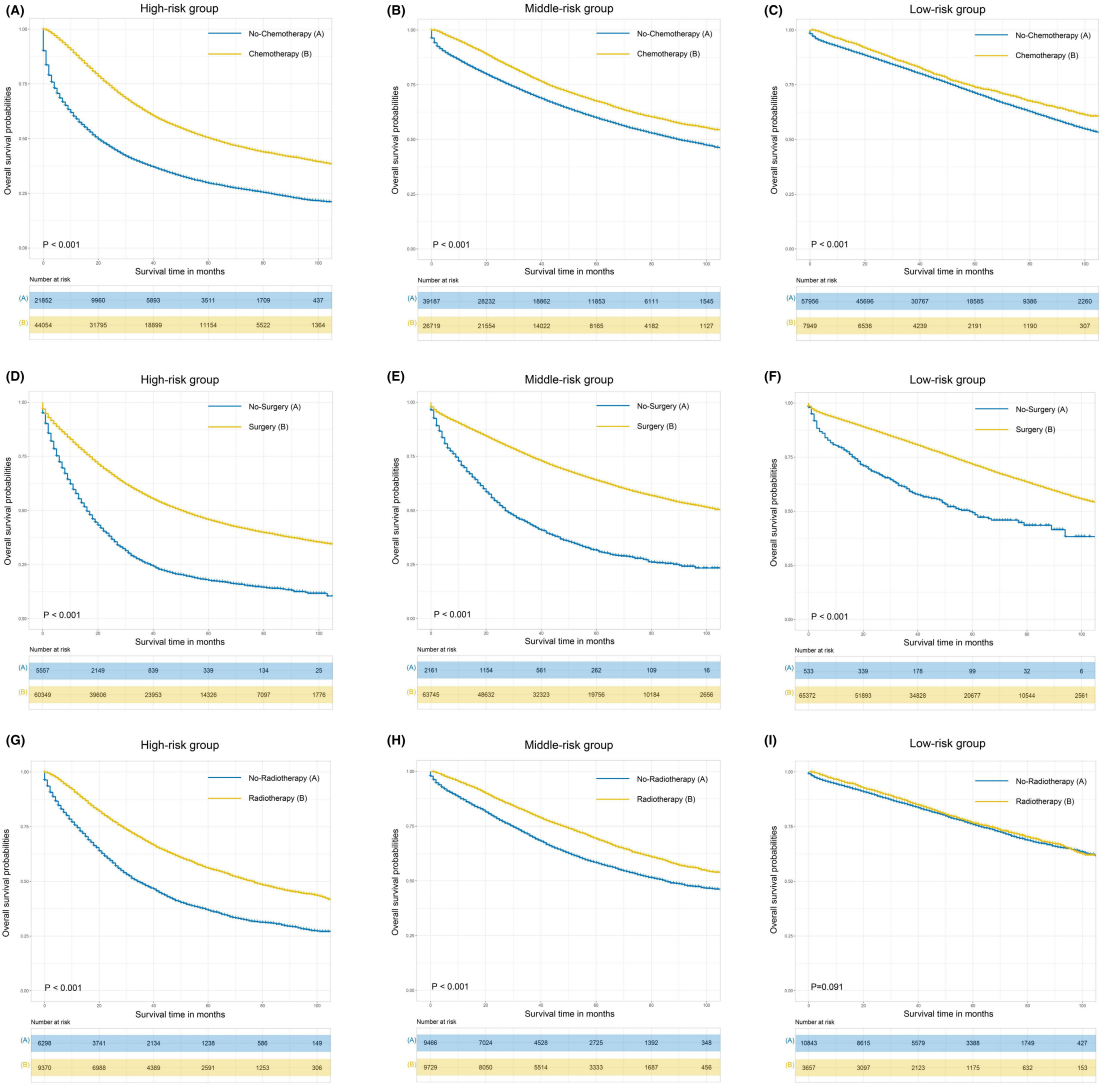
The OS comparison between CRC patients who received surgery and those who did not undergo surgery in the high‐risk group (A), middle‐risk group (B), and low‐risk group (C). The OS comparison between CRC patients who received chemotherapy and those who did not receive chemotherapy in the high‐risk group (D), middle‐risk group (E), and low‐risk group (F). The OS comparison between CRC patients who received radiotherapy and those who did not receive radiotherapy in the high‐risk group (G), middle‐risk group (H), and low‐risk group (I). CRC, colorectal cancer; OS, overall survival.

Using propensity score matching based on age and year of CRC diagnosis, race, sex, T stage, N stage, tumor size, and histology, we balanced the clinicopathological features of patients with and without treatment to assess the benefit of treatment (surgery, chemotherapy, and radiotherapy) for patients with CRC in the three risk score groups. Furthermore, we examined the OS of patients with balanced baseline characteristics in the three risk groups (Figure 6A–I, Figure S1). The results revealed that surgery, chemotherapy, and radiotherapy could significantly improve OS for CRC patients with CRC in the high‐ and middle‐risk groups. However, patients in the low‐risk group only benefited from surgery and chemotherapy whereas, concerning OS, radiotherapy failed to improve survival benefits.

## 4 DISCUSSION

As a relatively aggressive disease, approximately 20% of CRC patients developed DM at the initial diagnosis, which can lead to treatment failure and poor prognoses. Hence, timely diagnosis and treatment for metastases from CRC are expected to improve clinical outcomes. As previously mentioned, the liver and lung are the most common metastatic sites. Therefore, liver and lung imaging examinations are routinely implemented to detect hepatic and pulmonary metastases during the primary diagnosis of CRC. Moreover, despite rarities, the incidence of bone and brain metastases is increasing due to the development of multidisciplinary treatment and prolonged survival of patients with CRC. The bone is a common organ for DM from solid tumors, including malignancies in the prostate, breast, thyroid, lung, and kidney, while bone metastases from CRC are relatively rare.^24^, ^25^ Because of low incidence, relevant studies are lacking, and the therapeutic standards for bone and brain metastases have not yet been established. In clinical practice, the diagnosis of bone metastases from CRC relies on the manifestation of acute complications of bone destruction. Similarly, brain imaging is not conventionally performed to detect brain metastases until the onset of neurological symptoms. Knowledge about the early detection of DM (especially for bone and brain metastases) remains insufficient, and potential predictive risk factors are poorly understood. Our study demonstrated that N stage, T stage, tumor size and site, and amount of harvested lymph nodes were important risk factors for DM (including metastases to bone and brain). Previous studies have reported risk factors favoring bone metastases and risk prediction models, which was partially in line with results of our study. Sun et al. revealed that the tumor site and lymph node invasion contributed to bone metastases in CRC patients after curative resection.^26^ Moreover, a scoring system that comprised several factors, including rectal cancer, poor differentiation, CEA positivity, and metastases to lymph nodes and ectosteal organs, was constructed to predict the likelihood of bone metastases for patients newly diagnosed with CRC.^27^ Additionally, another scoring system that predicted the risk of developing bone metastases in CRC patients who received radical resection was reported, which could divide patients into different risk groups according to three independent risk factors, namely, pulmonary metastases, lymph node metastases, and rectal cancer.^28^ Han et al. found that tumor location, grade, T/N stage, and other factors were correlated with the occurrence of bone metastases and then developed a nomogram based on these factors to predict bone metastases.^29^ In the cases of brain metastases, studies on risk factors remain limited. Michl et al. conducted a study with the largest number of CRC patients who had brain metastases, revealed that brain metastases were the last event in the disease and tumor location was correlated with brain metastases. Moreover, the primary tumors of patients with brain metastases were predominantly left‐sided, particularly in the rectum.^30^ Other studies also demonstrated that brain metastases occurred more commonly in patients with primary left‐sided tumors.^31^ Quan et al. reported that most patients with brain metastases from CRC also had concomitant metastases in lungs, liver, and bone. Among these extracranial metastases, the lung was the most common site.^32^ Although liver metastases account for 70% of DM in metastatic CRC patients, liver metastases were not correlated with a higher risk of brain metastases, and some studies even found that patients with hepatic metastases had lower incidence of brain metastases than those with pulmonary metastases.^33^, ^34^, ^35^ In addition, Lu et al. utilized the deep learning model based on MRI data to accurately assess pelvic lymph node metastasis in rectal patients, which was better than the diagnosis and identification of radiologists in terms of both diagnostic quality and speed.^36^ However, the application of radiomics is based on the hypothesis that a large amount of information from images can be extracted by radiomics, translating macroscopic image‐based features into pathologic information. In radiomics studies, a large number of unexplainable radiomics features were extracted from images, most of which lack clinical significance. Most existing predictive models developed using complex and diverse radiomics data features are extremely difficult to reproduce and popularize in clinical practice.^37^ Furthermore, the wide range of imaging protocols, scanner types, and diagnostic criteria for tumor metastases affects the accuracy, leading to highly heterogeneous results.

In the present study, we used clinicopathological data from the NCC and SEER database to establish the first risk model using RF to predict the probability of DM (including liver, lung, and bone and brain metastases) in patients newly diagnosed with CRC. We found that N stage, T stage, tumor size, harvested lymph nodes, age at diagnosis, and tumor site played vital roles in this clinicopathological characteristic‐based risk model, which partially accorded with the aforementioned studies. Note that the similar risk models and nomograms for predicting CRC metastases reported in several studies were mainly based on multivariable logistic regression, and there is no machine learning‐based risk model applied to identify patients at high risk of developing DM so far, particularly bone and brain metastases.^27^, ^28^, ^29^ Thus, we have developed precise risk models using logistic regression and different machine learning algorithms, such as RF, XGboost, DNN, and KNN. It has been shown that RF model exhibited the highest AUC in all cohorts, suggesting that RF‐based prediction model had better discrimination than other machine learning algorithms and logistic regression, which could accurately identify patients who probably need further costly examinations (such as PET‐CT) to detect potential distant metastases. Moreover, the previously reported risk models and nomograms could merely forecast single‐site metastases in patients with CRC.^27^, ^28^, ^29^ Remarkably, the RF‐based risk model in this study can precisely evaluate the likelihood of multiple DM from CRC, including liver, lung, and bone and brain metastases, offering physicians a more convenient predictive tool to identify patients with a higher risk of DM during primary diagnosis. Furthermore, according to the DM risk score obtained from our risk model, we can divide patients newly diagnosed with CRC into the high‐, middle‐, and low‐risk groups. Patients in the high‐risk group had a much greater possibility of developing DM at the primary CRC diagnosis, alerting physicians to promptly detect metastases through intensive screening modalities, such as PET–CT and neurological imaging, even though these are not recommended by clinical guidelines. For patients in the middle and low‐risk groups, routine follow‐up might be appropriate. Therefore, with this predictive clinical tool, physicians can evaluate the risk and improve surveillance managements for patients according to their DM risk score. Moreover, the DM‐risk stratification based on the risk model in this study first analyzed the efficacy of different treatment regimens for patients with CRC by comparing survival outcomes of patients receiving different treatments in three risk‐level groups. It was shown that surgery, chemotherapy, and radiotherapy were beneficial for patients in high and middle‐risk groups. Consistent with the results of our study, previous studies found that surgery or surgery plus radiotherapy was related to better survival outcomes in patients with CRC with brain metastases.^38^ For patients in the low‐risk group, surgery and chemotherapy could improve prognosis, while radiotherapy was not correlated with better survival outcomes, suggesting that radiotherapy might not be an ideal treatment option for patients in the low‐risk group and could cause unnecessary radiation‐induced injuries.

To date, this is the first study to build a risk model using multiple machine learning algorithms to predict the likelihood of developing multi‐organ metastasis in newly diagnosed CRC patients. The large sample size (197,717 patients from SEER and 3241 patients from the NCC) was a distinctive advantage of this population‐based study. Moreover, we used the external NCC cohort to validate the prediction model. This RF‐based risk model exhibited good accuracy for predicting DM in all cohorts, which can be regarded as another strength. Furthermore, in view of clinical practicability, the risk factors in this risk model, including T/N stage, tumor size, tumor site, and age at diagnosis, are accessible clinicopathological characteristics, which can be easily obtained in routine clinical practice, thus helping physicians predict the risk of metastases and take more individualized examinations and surveillance strategies. Our prediction models could assess the benefits of surgery, chemotherapy, and radiotherapy for CRC patients belonging to different risk score groups, which could assist clinicians to select optimal treatment.

However, there were following shortcomings must be acknowledged in our study. First, the retrospective nature of this study should be noted, and the absence of some clinicopathological factors inevitably resulted in bias. Additionally, because the patients involved in this study could only represent the American and Chinese populations, further study recruiting patients from more countries is needed. Besides, the lack of genetic information in the SEER database, such as RAS/BRAF/MSI mutation status, which could improve the accuracy and use of the risk model, is another disadvantage of this study. And family history of cancer might be a potential cofounding factor, thus we collected the patients without family history of cancer in the external validation cohort from the NCC to avoid bias. However, there was no detailed information about family history of cancer for patients from the SEER database, which was a shortcoming for our study. Last but not least, the SEER database lacked a detailed record of patient‐specific chemotherapy regimens. The application of chemotherapy with different regimens and courses might have an influence on both prognosis and DM, thus validation of the influence brought by different chemotherapy regimens for DM risk and survival outcomes should be further conducted. Meanwhile, the time to surgery and adjuvant therapy can definitely have an impact on DM, which also was lacking in the SEER database necessitating further analysis. As for patients in external validation set from the NCC, the chemotherapy regimens for them included Xelox (Oxaliplatin and Capecitabine) and FOLFOX (5‐Fluorouracil, Calcium Folinate, and Oxaliplatin). There was no delay in the time to surgery and adjuvant therapy of patients who recruited in the external set. Despite these limitations, this risk model and stratification remain a useful clinical tool for predicting the possibility of DM at the initial diagnosis of CRC and providing guidance for clinical decision‐making.

## 5 CONCLUSION

In summary, using clinicopathological data from the NCC and SEER databases, we established a novel RF‐based risk model to predict the possibility of metastases to multiple organs, including the liver, lungs, bone, and brain, in patients with newly diagnosed CRC. According to the risk of DM, this prediction model could stratify patients into high‐, middle‐, and low‐risk groups, which could assist physicians in identifying patients with high risk of developing DM and thus optimize therapeutic management.

## AUTHOR CONTRIBUTIONS

**Ran Wei:** Data curation (equal); formal analysis (equal); investigation (equal); methodology (equal); software (equal); validation (equal); visualization (equal); writing – original draft (equal); writing – review and editing (equal). **Guanhua Yu:** Data curation (equal); formal analysis (equal); investigation (equal); methodology (equal); software (equal); validation (equal); visualization (equal); writing – original draft (equal); writing – review and editing (equal). **Xishan Wang:** Project administration (equal); resources (equal). **Zheng Jiang:** Project administration (equal); resources (equal); supervision (equal). **Xu Guan:** Conceptualization (equal); project administration (equal); resources (equal); supervision (equal).

## FUNDING INFORMATION

None.

## CONFLICT OF INTEREST STATEMENT

The authors declare that the research was conducted in the absence of any commercial or financial relationships that could be construed as a potential conflict of interest.

## CONSENT FOR PUBLICATION

All authors consent for publication.

## ETHICS APPROVAL AND CONSENT TO PARTICIPATE

The study was conducted in accordance with the Declaration of Helsinki and approved by the Ethics Committee of the National Cancer Center/National Clinical Research Center for Cancer/Cancer Hospital, Chinese Academy of Medical Sciences (NCC2019S‐060). The study did not require written informed consent for retrospective analysis of de‐identified and anonymized data.

## Supporting information


Data S1.

## Data Availability

The data of training cohort and test cohort analyzed in the present study are available in the Surveillance, Epidemiology, and End Results (SEER) database (https://seer.cancer.gov/data/). The data of external validation cohort from the National Cancer Center/National Clinical Research Center for Cancer/Cancer Hospital, Chinese Academy of Medical Sciences, and Peking Union Medical College will be shared on reasonable request to the corresponding author.
