# Supplementary material for: Construction and validation of machine learning models for predicting distant metastases in newly diagnosed colorectal cancer patients: A large‐scale and real‐world cohort study
Source: Cancer Med. 2024 Mar 16;13(5):e6971. doi: 10.1002/cam4.6971 (PMC10943273; doi:10.1002/cam4.6971)
Supplement: Supplementary file 1 — Data S1. [file CAM4-13-e6971-s001.docx]

**Supplementary Table 1-11:**

**Supplementary Table 1.** Demographic and tumor characteristics of patients with colorectal cancer in SEER database.

| **Characteristic** | **SEER database** | | |
| --- | --- | --- | --- |
|  | **Non-Metastasis (n=177674)** | **Metastasis (n=20043)** | **P-Value** |
| **Age at CRC diagnosis** |  |  | <0.001 |
| 20-49 | 18605 (10.5) | 3168 (15.8) |  |
| 50-69 | 77750 (43.8) | 10013 (50.0) |  |
| ≥ 70 | 81319 (45.7) | 6862 (34.2) |  |
| **Gender** |  |  | <0.001 |
| Female | 86675 (48.8) | 8808 (43.9) |  |
| Male | 90999 (51.2) | 11235 (56.1) |  |
| **Year of CRC diagnosis** |  |  | <0.001 |
| 2010-2013 | 88094 (49.6) | 10446 (52.1) |  |
| 2014-2018 | 89580 (50.4) | 9597 (47.9) |  |
| **Race, No. (%)** |  |  | <0.001 |
| White | 141399 (79.6) | 15233 (76.0) |  |
| Black | 19804 (11.1) | 2980 (14.9) |  |
| Other | 16471 (9.3) | 1830 (9.1) |  |
| **Grade, No. (%)** |  |  | <0.001 |
| Grade1/2 | 145172 (81.7) | 14542 (72.6) |  |
| Grade3/4 | 32502 (18.3) | 5501 (27.4) |  |
| **Primary tumor site** |  |  | <0.001 |
| Right Colon | 86497 (48.6) | 8789 (43.9) |  |
| Left Colon | 46830 (26.4) | 6225 (31.1) |  |
| Rectum | 44347 (25.0) | 5029 (25.0) |  |
| **T stage** |  |  | <0.001 |
| T1/T2 | 55824 (31.4) | 2068 (10.3) |  |
| T3/T4 | 121850 (68.6) | 17975 (89.7) |  |
| **N stage** |  |  | <0.001 |
| N0 | 108169 (60.9) | 4406 (22.0) |  |
| N1/N2 | 69505 (39.1) | 15637 (78.0) |  |
| **Tumor histology** |  |  | <0.001 |
| Adenocarcinomas | 122419 (68.9) | 16022 (79.9) |  |
| Other | 55255 (31.1) | 4021 (20.1) |  |
| **Tumor size** |  |  | <0.001 |
| (0-2] | 32076 (18.1) | 995 (5.0) |  |
| (2-5] | 88615 (49.9) | 9679 (48.3) |  |
| 5< | 56983 (32.0) | 9369 (46.7) |  |
| **Number of nodes examined** |  |  | <0.001 |
| >12 | 140747 (79.2) | 13947 (69.6) |  |
| <12 | 36927 (20.8) | 6096 (30.4) |  |
| **Surgery** |  |  |  |
| No | 5293 (2.9) | 3467 (6.6) | <0.001 |
| Yes | 172381 (97.1) | 48287 (93.3) |  |
| **Chemotherapy** |  |  | <0.001 |
| No | 113031 (63.6) | 5964 (29.8) |  |
| Yes | 64643 (36.4) | 14079 (70.2) |  |
| **Radiation** |  |  | 0.003 |
| No | 154677 (87.1) | 17597 (87.8) |  |
| Yes | 22997 (12.9) | 2446 (12.2) |  |
| **Primary tumor** |  |  | <0.001 |
| Yes | 138579 (78.0) | 16728 (83.5) |  |
| No | 39095 (22.0) | 3315 (16.5) |  |

**NOTE.** P values were calculated using the χ2 test for categorical variables.

**Abbreviations:** CRC, colorectal cancer; SEER, Surveillance, Epidemiology, and End Results.

**Supplementary Table 2.** Performance of machine learning models.

|  | **Training cohort** | | | | **Test cohort** | | | | **External validation cohort** | | | |
| --- | --- | --- | --- | --- | --- | --- | --- | --- | --- | --- | --- | --- |
|  | **AUC (95%CI)** | **Gini index** | **1 - Specificity** | **Sensitivity** | **AUC (95%CI)** | **Gini index** | **1 - Specificity** | **Sensitivity** | **AUC (95%CI)** | **Gini index** | **1 - Specificity** | **Sensitivity** |
| **Random Forest** | 0.843 (0.840-0.846) | 0.687 | 0.841 | 0.687 | 0.793 (0.787-0.801) | 0.587 | 0.802 | 0.652 | 0.806 (0.788-0.833) | 0.612 | 0.750 | 0.715 |
| **Extreme Gradient Boosting** | 0.802 (0.798-0.805) | 0.604 | 0.809 | 0.658 | 0.788 (0.781-0.794) | 0.576 | 0.837 | 0.611 | 0.785 (0.768-0.803) | 0.571 | 0.886 | 0.813 |
| **Deep Neural Network** | 0.776 (0.772-0.779) | 0.552 | 0.785 | 0.647 | 0.774 (0.767-0.781) | 0.549 | 0.778 | 0.638 | 0.724 (0.706-0.742) | 0.428 | 0.816 | 0.555 |
| **Logistic Regression** | 0.797 (0.794-0.801) | 0.595 | 0.839 | 0.623 | 0.794 (0.787-0.801) | 0.588 | 0.827 | 0.63 | 0.785 (0.767-0.802) | 0.570 | 0.632 | 0.816 |
| **K-nearest neighbor** | 0.820 (0.817-0.824) | 0.641 | 0.745 | 0.768 | 0.723 (0.714-0.731) | 0.446 | 0.678 | 0.61 | 0.759 (0.743-0.776) | 0.519 | 0.629 | 0.798 |

**NOTE.** Accuracy of postoperative complications prediction for the Random Forest, Extreme Gradient Boosting, Deep Neural Network, Support Vector Machine, Logistic Regression and K-nearest neighbor in training set, testing set and external validation cohort.

**Supplementary Table 3.** The DeLong test for AUC of machine learning models in training cohort.

|  | **Random Forest** | **Extreme Gradient Boosting** | **Deep Neural Network** | **Logistic Regression** | **K-nearest neighbor** |
| --- | --- | --- | --- | --- | --- |
| **Random Forest** | NA | <0.001 | <0.001 | <0.001 | <0.001 |
| **Extreme Gradient Boosting** | NA | NA | >0.05 | <0.001 | <0.001 |
| **Deep Neural Network** | NA | NA | NA | <0.001 | <0.001 |
| **Logistic Regression** | NA | NA | NA | NA | <0.001 |
| **K-nearest neighbor** | NA | NA | NA | NA | NA |

**NOTE.** The DeLong test for AUC of Random Forest, Extreme Gradient Boosting, Deep Neural Network, Support Vector Machine, Logistic Regression and K-nearest neighbor in training set.

**Supplementary Table 4.** The DeLong test for AUC of machine learning models in testing cohort.

|  | **Random Forest** | **Extreme Gradient Boosting** | **Deep Neural Network** | **Logistic Regression** | **K-nearest neighbor** |
| --- | --- | --- | --- | --- | --- |
| **Random Forest** | NA | <0.01 | >0.05 | <0.001 | <0.001 |
| **Extreme Gradient Boosting** | NA | NA | <0.01 | <0.001 | <0.001 |
| **Deep Neural Network** | NA | NA | NA | <0.001 | <0.001 |
| **Logistic Regression** | NA | NA | NA | NA | <0.001 |
| **K-nearest neighbor** | NA | NA | NA | NA | NA |

**NOTE.** The DeLong test for AUC of Random Forest, Extreme Gradient Boosting, Deep Neural Network, Support Vector Machine, Logistic Regression and K-nearest neighbor in testing set.

**Supplementary Table 5.** The DeLong test for AUC of machine learning models in validation cohort.

|  | **Random Forest** | **Extreme Gradient Boosting** | **Deep Neural Network** | **Logistic Regression** | **K-nearest neighbor** |
| --- | --- | --- | --- | --- | --- |
| **Random Forest** | NA | <0.001 | <0.001 | <0.001 | <0.001 |
| **Extreme Gradient Boosting** | NA | NA | >0.05 | <0.001 | <0.001 |
| **Deep Neural Network** | NA | NA | NA | <0.001 | <0.001 |
| **Logistic Regression** | NA | NA | NA | NA | <0.001 |
| **K-nearest neighbor** | NA | NA | NA | NA | NA |

**NOTE.** The DeLong test for AUC of Random Forest, Extreme Gradient Boosting, Deep Neural Network, Support Vector Machine, Logistic Regression and K-nearest neighbor in validation cohort.

**Supplementary Table 6.** The feature importance for predicting postoperative complications of Extreme Gradient Boosting.

| **Factors** | **Importance (%)** |
| --- | --- |
| N stage | 18.93 |
| T stage | 17.09 |
| Age at diagnosis | 9.85 |
| Harvested lymph node | 9.49 |
| Tumor size | 8.60 |
| Tumor site | 7.66 |
| Race | 7.29 |
| Grade | 5.96 |
| Gender | 4.09 |
| Year at diagnosis | 4.02 |
| Histologic type | 3.91 |
| Primary cancer | 3.03 |

**Supplementary Table 7.** The feature importance for predicting postoperative complications of Logistic Regression.

| **Factors** | **Importance (%)** |
| --- | --- |
| Age at diagnosis | 9.15 |
| Gender | 4.19 |
| Year at diagnosis | 2.04 |
| Race | 0.57 |
| Tumor site | 3.63 |
| Histologic type | 5.97 |
| Grade | 3.19 |
| Harvested lymph node | 19.45 |
| Primary cancer | 2.44 |
| T stage | 10.15 |
| N stage | 31.01 |
| Tumor size | 8.15 |

**Supplementary Table 8.** The feature importance for predicting postoperative complications of Deep Neural Network.

| **Factors** | **Importance (%)** |
| --- | --- |
| Age at diagnosis | 11.73 |
| Gender | 5.70 |
| Year at diagnosis | 4.41 |
| Race | 7.03 |
| Tumor site | 9.09 |
| Histologic type | 6.44 |
| Grade | 5.19 |
| Harvested lymph node | 11.76 |
| Primary cancer | 4.77 |
| T stage | 10.86 |
| N stage | 12.43 |
| Tumor size | 10.53 |

**Supplementary Table 9.** The feature importance for predicting postoperative complications of K-nearest neighbor.

| **Factors** | **Importance (%)** |
| --- | --- |
| N stage | 45.79 |
| T stage | 22.72 |
| Tumor size | 12.14 |
| Age at diagnosis | 4.51 |
| Histologic type | 4.32 |
| Grade | 4.06 |
| Harvested lymph node | 3.85 |
| Primary cancer | 1.29 |
| Gender | 0.71 |
| Tumor site | 0.23 |
| Year at diagnosis | 0.18 |
| Race | 0.15 |

**Supplementary Table 10.** The feature importance for predicting postoperative complications of random forest.

| **Factors** | **Importance (%)** |
| --- | --- |
| Age at diagnosis | 7.09 |
| Gender | 3.89 |
| Year at diagnosis | 3.97 |
| Race | 6.16 |
| Tumor site | 6.97 |
| Histologic type | 3.95 |
| Grade | 3.65 |
| Harvested lymph node | 7.36 |
| Primary cancer | 3.41 |
| T stage | 17.43 |
| N stage | 27.22 |
| Tumor size | 8.84 |

**Supplementary Table 11.** Demographic and tumor characteristics of patients with colorectal cancer among three different risk groups.

| **Characteristic** | **High-risk group**  **(n=65906)** | **Middle-risk group**  **(n=65906)** | **Low-risk group**  **(n=65905)** | **P-Value** |
| --- | --- | --- | --- | --- |
| **Age at CRC diagnosis, No. (%)** |  |  |  | <0.001 |
| 20-49 | 11292 (17.1) | 6754 (10.2) | 3727 (5.7) |  |
| 50-69 | 34037 (51.6) | 28179 (42.8) | 25547 (38.8) |  |
| ≥ 70 | 20577 (31.3) | 30973 (47.0) | 36631 (55.5) |  |
| **Gender, No. (%)** |  |  |  | <0.001 |
| Female | 28351(43.0) | 32789 (49.8) | 34344 (52.1) |  |
| Male | 37555 (57.0) | 33117 (50.2) | 31562 (47.9) |  |
| **Year of CRC diagnosis, No. (%)** |  |  |  | <0.001 |
| 2010-2013 | 35079 (53.2) | 32880 (49.9) | 30581 (46.4) |  |
| 2014-2018 | 30827 (46.8) | 33026 (50.1) | 35324 (53.6) |  |
| **Race, No. (%)** |  |  |  | <0.001 |
| White | 50411 (76.5) | 50934 (77.3) | 55287 (83.9) |  |
| Black | 9809 (14.9) | 7897 (12.0) | 5078 (7.7) |  |
| Other | 5686 (8.6) | 7075 (10.7) | 5540 (8.4) |  |
| **Grade, No. (%)** |  |  |  | <0.001 |
| Grade1/2 | 46056 (69.9) | 55089 (83.6) | 58569 (88.9) |  |
| Grade3/4 | 19850 (30.1) | 10817 (16.4) | 7336 (11.1) |  |
| **Primary tumor site, No. (%)** |  |  |  | <0.001 |
| Right Colon | 29917 (45.4) | 25694 (39.0) | 39675 (60.2) |  |
| Left Colon | 20413 (31.0) | 20940 (31.8) | 11702 (17.8) |  |
| Rectum | 15576 (23.6) | 19272 (29.2) | 14528 (22.0) |  |
| **T stage, No. (%)** |  |  |  | <0.001 |
| T1/T2 | 4224 (6.4) | 14477 (22.0) | 39191 (59.5) |  |
| T3/T4 | 61682 (93.6) | 51429 (78.0) | 26714 (40.5) |  |
| **N stage, No. (%)** |  |  |  | <0.001 |
| N0 | 6574 (10.0) | 42768 (64.9) | 63233 (95.9) |  |
| N1/N2 | 59332 (90.0) | 23138 (35.1) | 2672 (4.1) |  |
| **Tumor histology, No. (%)** |  |  |  | <0.001 |
| Adenocarcinomas | 53507 (81.2) | 47216 (71.6) | 37718 (57.2) |  |
| Other | 12399 (18.8) | 18690 (28.4) | 28187 (42.8) |  |
| **Tumor size, No. (%)** |  |  |  | <0.001 |
| (0-2] | 1409 (2.1) | 10624 (16.1) | 21038 (31.9) |  |
| (2-5] | 31395 (47.6) | 34944 (53.0) | 31955 (48.5) |  |
| >5 | 33102 (50.3) | 20338 (30.9) | 12912 (19.6) |  |
| **Number of nodes examined, No. (%)** |  |  |  | <0.001 |
| <12 | 47239 (71.7) | 51612 (78.3) | 55843 (84.7) |  |
| >=12 | 18667 (28.3) | 14294 (21.7) | 10062 (15.3) |  |
| **Chemotherapy, No. (%)** |  |  |  | <0.001 |
| No | 21852 (33.2) | 39187 (59.5) | 57956 (87.9) |  |
| Yes | 44054 (66.8) | 26719 (40.5) | 7949 (12.1) |  |
| **Radiation, No. (%)** |  |  |  | <0.001 |
| No | 54981 (83.4) | 55293 (83.9) | 62000 (94.1) |  |
| Yes | 10925 (16.6) | 10613 (16.1) | 3905 (5.9) |  |
| **Primary tumor, No. (%)** |  |  |  | <0.001 |
| Yes | 56211 (85.3) | 50722 (77.0) | 48374 (73.4) |  |
| No | 9695 (14.7) | 15184 (23.0) | 17531 (26.6) |  |
| **CRC metastatic, No. (%)** |  |  |  | <0.001 |
| No | 48298 (73.3) | 60847 (92.3) | 64717 (98.2) |  |
| Yes | 17608 (26.7) | 5059 (7.7) | 1188 (1.8) |  |

**Supplementary Figure:**


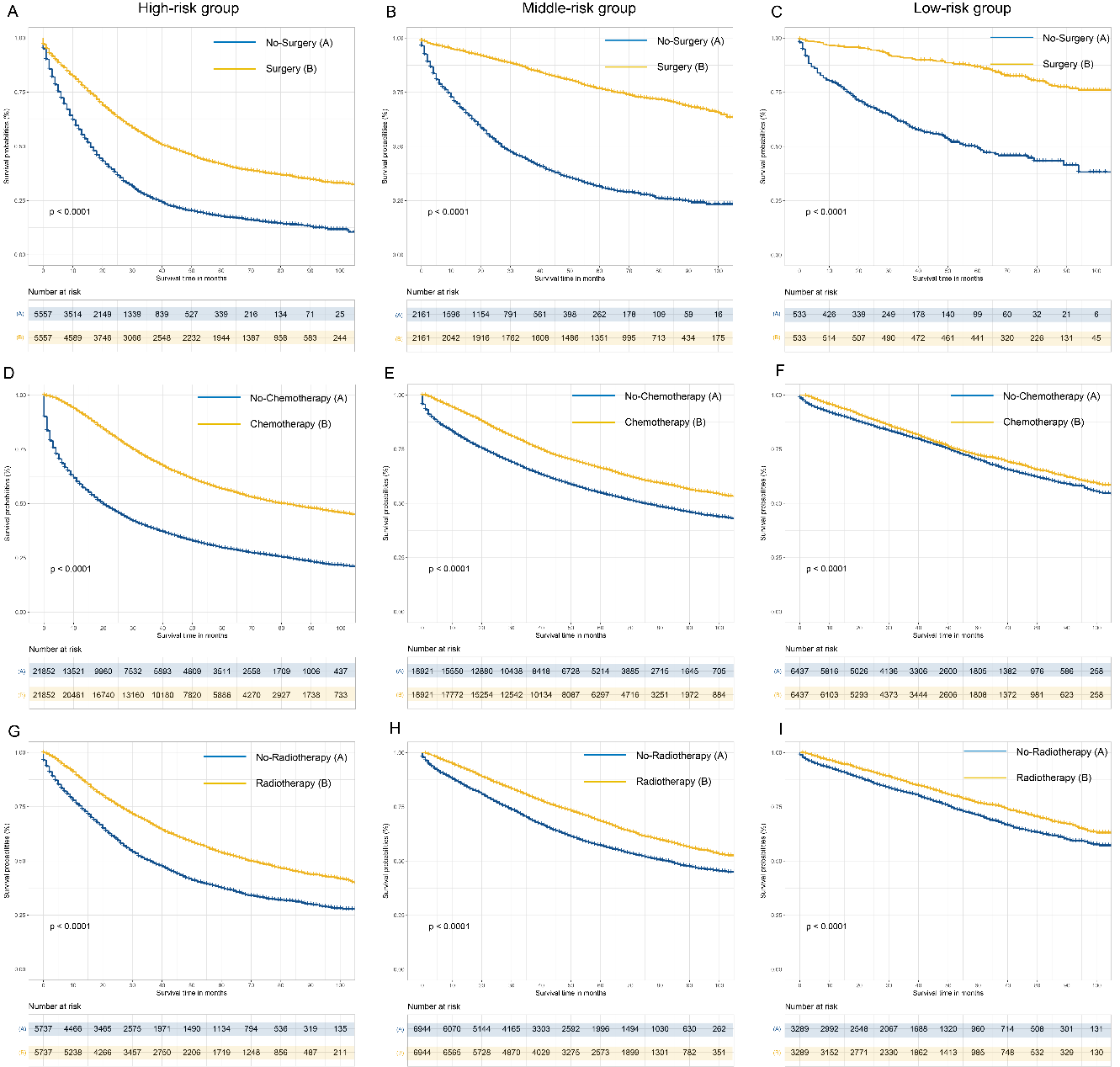


**Supplementary Figure 1.** The overall survival (OS) comparison between colorectal cancer (CRC) patients who received surgery and those who did not undergo surgery in the high-risk group (A), middle-risk group (B) and low-risk group (C). Survival comparison between patients with CRC who received chemotherapy and those who did not receive chemotherapy in the high-risk group (D), middle-risk group (E) and low-risk group (F). Survival comparison between patients with CRC who received radiotherapy and those who did not receive radiotherapy in the high-risk group (G), middle-risk group (H) and low-risk group (I).

**NOTE.** The CRC patients who receive treatment (including surgery, chemotherapy and radiotherapy) and non-treatment at a PSM ratio of 1:1. (A, B, C) CRC patients who receive surgery and CRC patients who didn’t receive surgery were matched by PSM at a ratio of 1:1. (D, E, F) CRC patients who receive chemotherapy and PC patients who didn’t receive chemotherapy were matched by PSM at a ratio of 1:1. (G, H, I) CRC patients who receive radiotherapy and CRC patients who didn’t receive radiotherapy were matched by PSM at a ratio of 1:1.The matched variables for propensity score matching (PSM) included the age at CRC diagnosis, race, gender, T stage, N stage, year of CRC diagnosis, tumor size and histology at the ratio of 1:1.
